# Assessment of pulmonary function among woodworkers exposed to mixed tropical hardwood dust in Kumasi, Ghana

**DOI:** 10.4314/ahs.v25i4.16

**Published:** 2025-12

**Authors:** Isaac Ekow Ennin, Margaret Agyei Frempong, Francis A Yeboah, Raymond Saa-Eru Maalman

**Affiliations:** 1 Department of Physician Assistantship, School of Medicine and Health Sciences, Central University Accra, Greater Accra Region, Ghana; 2 Department of Molecular Medicine, School of Medical Science, Kwame Nkrumah University of Science and Technology, Kumasi, Ashanti Region, Ghana; 3 Department of Basic Medical Sciences, School of Medicine, University of Health and Allied Sciences, Ho Volta Region, Ghana

**Keywords:** Lung function, Respiratory symptoms, Mixed tropical hardwood dust and woodworkers

## Abstract

**Introduction:**

Several studies have shown that woodworkers present with frequent respiratory symptoms and reduced lung volume and airflow values, including FVC, FEV1, FEV1/FVC, and PEFR, than controls from the general population. However, other studies have reported no significant negative health effects of wood dust on the respiratory system. The effect of wood dust on pulmonary function depends on the wood species, phytochemicals present in the wood, and the concentration level of ambient air wood dust. The ambient air dust concentration level at the wood workshops may depend on the humidity and ventilation at the workshops. This study aimed at assessing the pulmonary function of woodworkers exposed to mixed tropical hardwoods in Kumasi, Ghana.

**Method:**

The study was conducted among woodworkers, teachers, and security men located in Kumasi. A cross-sectional, clustered, and convenient sample of 195 adult male workers (96 woodworkers and 99 non-wood workers) were enrolled for the study. Pulmonary function assessment was carried out using an adapted version of the British Medical Research Council's (MRC) questionnaire on respiratory symptoms and apirometer (Alpha Vitalograph) for lung function testing. The relative humidity and the level of wood dust exposure to woodworkers were measured using the personal exposure meter (PATS+: Berkeley Air Monitoring Group) from the Environmental Protection Authority.

**Results:**

The mean percentage relative humidity in the workshop was 68%, and the ambient air wood dust concentration ranged between 0.003 and 1.02 mg/m^3^. Respiratory symptoms prevalent at the workplace among woodworkers ranged between 8-76% (p<0.05). The VC and FVC values were significantly low in the woodworkers (p>0.05).

**Conclusion:**

The study revealed that woodworkers exposed to mixed tropical hardwood dust without personal protection equipment showed an increase in the prevalence of rhinitis and sneezing and a significant reduction in the VC and FVC values at the workplace.

## Introduction

High levels of wood dust exposure among woodworkers have been suggested to cause occupational pulmonary disorders^1^. Woodworkers exposed to wood dust levels ranging from 0.2 to 7.4 mg/m^3^ have suffered various respiratory symptoms^2^. Bohadana et al.^3^ reported bronchial hyper-reactivity in woodworkers exposed to oak and beech dust levels of between 2.96–12.74 mg/m^3^.

A study at the Accra Timber Market reported on significantly reduced lung volumes and airflow values in workers exposed to tropical hardwood dust levels ranging between 0.19 - 0.71 mg/m3^4^. In another study, Tobin et al.^5^ reported a high prevalence of respiratory symptoms, particularly cough and phlegm, among tropical hardwood workers with a mean exposure level of 1.39 mg/m^3^. Earlier epidemiological studies have shown that exposure to both softwood and hardwood dust is associated with several non-malignant health effects such as allergic rhinitis, chronic bronchitis, and allergic asthma^6^.

Respiratory disorders have been displayed in spirometry as reduced lung function^7^,^8^. Many studies have shown that exposed woodworkers have reduced lung function parameters including VC, FVC, FEV1, FEV1/FVC, and PEFR compared to controls from the general population^9^-^12^. However, a number of studies have reported no significant negative health effects of wood dust on the respiratory system. In their study, Fransman et al.^13^ did not find any relationship between wood dust and respiratory symptoms such as rhinitis. Nde et al.^14^, in another study among woodworkers exposed to tropical hardwood dust, reported no significant difference between the prevalence of abnormalities in lung function of carpenters and the matched non-exposed group. To confirm the role of exposure to tropical hardwood dust in the respiratory system, the present study was conducted to determine the frequency of respiratory symptoms and the status of lung function at the workplace. In addition, the ambient air, wood dust, and humidity of the workplace were determined.

## Materials and Methods

### Definitions

Frequent respiratory symptoms were defined in this study as respiratory symptoms that manifested every day at the work place.

Rhinitis was defined in this study as the occurrence of frequent sneezing, coughing, itching nose, blocked nose, and runny nose (Catarrh) in a participant.

Vacation is defined in our study as two or more days away from a work place.

### Study sites and population

A cross-sectional survey was conducted to investigate the pulmonary function of workers exposed to tropical hardwood in Sokoban Wood Village, Kumasi. The wood village has a population of about 6000 including other traders in the wood village^15^. Kumasi has an average annual rainfall of 1,270 mm and two rainy seasons, an average daily temperature of about 27°C and the average annual percentage of humidity is 75% (Average Humidity in Kumasi, 2021).

A sawmill shop in the Sokoban Wood Village occupies an area of about 18-36m^2^ with high rise concrete pillars and roofing to improve ventilation and create shelter, respectively. About 6 to 8 shops are adjoined to each other in a chain. The shops do not have walls enclosing them from floor to roof; however, a few have short walls about one meter tall that divide one shop from the other. A shop may contain two or three different types of machines for different tasks, which generate different sizes of wood shavings and wood dust. A machine operator is not restricted to one type of machine. A shop may have 6-14 workers and a small office space occupied by a supervisor or a cashier. In each shop, machine operators, carpenters, a cashier, and a supervisor are found. Averagely work commences at 7:00 am and closes at 6:00 pm.

The timber species processed in the Sokoban wood village include Antiaris toxicaria, Khaya senegalensis, Piptadeniastum africanum, Celtis mildbraedii, Lophira alata, Ceiba pentandra, Triplochiton sclerexylon, Terminalia ivoresis, Terminalia superba, Melicia excels, Entandrophragma cylindricum, etc. The sawmills produce all kinds of wood products. The machines do not have wood dust extraction devices, often, the floors of the shops are littered with pieces of wood and heaps of wood shavings and dust which are collected and disposed of every morning to keep the working environment clean. Moreover, most of the woodworkers do not put on the nose masks, thus exposing themselves to inhalable wood dust. The participants comprising of 96 woodworkers, mainly machine operators and a carpenter selected from Sokoban Wood Village, volunteered to be subjects for the study. In addition, 99 non-exposed workers selected for control from the teachers of the Kwame Nkrumah University of Science and Technology (KNUST) Primary and Junior High Schools together with security staff of the KNUST Security Services in Kumasi were also recruited.

The study subjects were volunteers who satisfied the eligibility criteria; male workers between the ages of 20 - 60 years who have worked from 1 year and above without any clinical abnormalities of the vertebral column and the thoracic cage were included. They also must have no neuromuscular and respiratory disease, as well as no history of chest surgery, this was based on self-reported medical history. Woodworkers involved in machine operation and carpentry. Teachers and security men who have not been exposed to occupational air pollutants were the controls for the study.

The following were the exclusion criteria for the woodworkers; workers not willing to participate and workers with a history of smoking. Exclusion criteria for the controls were; workers previously not exposed to workplace air pollutants, based on self-reporting history.

### Sampling

#### Sample size determination

The sample size was determined using the statistical formula Ss = [z^2^ (p) (1-p)]/c^2^ considering the following assumptions, a confidence level of 1.96 for 95%, a prevalence rate of 2.0% and a confidence interval of 0.035 for a minimum sample size of 61. A total of 195 participants were included in the study.

#### Sampling technique

Convenience sampling was used at a durbar of the woodworkers in the wood village. The woodworkers were informed of the research and benefits. Interested workers were invited for recruitment and those who met the selection criteria were included in the study. The same process was used for the recruitment of the controls.

### Determination of ambient air wood dust

To determine the personal exposure to wood dust, machine operators and carpenters were selected due to their greater exposure to wood dust and working without any nose mask. The measurement of the ambient air wood dust concentration was done by Environmental Protection Agency (EPA) Technical personnel from Accra, and each machine was calibrated before being used. Two machine operators and two carpenters at about 100 meters intervals were sampled using personal exposure meters (PATS manufacture: Berkeley Air Monitoring Group) for ten hours of work. Each meter was strapped to the arms of the selected participants. Hourly recording was done for ten hours after which the meters were removed and sent to the EPA office in Accra for assessment of the dust exposure levels.

### Determination of Respiratory symptoms at the workplace

All participants completed a questionnaire on medical history, occupational history, and respiratory symptoms at the workplace and during vacation. A face-to-face interview in English or Twi was conducted by the principal investigator assisted by three National Service personnel who had first degrees in Medical Laboratory Technology and trained in data collection methods. Questions on respiratory symptoms were adapted from the Medical Research Council's (MRC) questionnaire on respiratory symptoms (1960). Components of the questionnaire on respiratory symptoms included variables such as frequency of sneezing, wheezing, dry cough, wet cough, fever, loss of voice, breathlessness at rest, breathlessness at work, chest pain and rhinitis (as defined under Section 2.2).

### Lung Function Testing

The test was performed using the electronic spirometer Vitalograph Alpha (Vitalograph LTD, Buckingham, England) according to the standards of (American Thoracic Society 2003). The analysis measured Vital Capacity (VC), Forced Vital Capacity (FVC), Forced Expiratory Volume in one second (FEV1), Forced Expiratory Volume ratio (FEV1/FVC) and Peak Expiratory Flow Rate (PEFR). The test had two manoeuvres; the static manoeuvre to measure VC and the dynamic manoeuvre to measure FVC, FEV1, FEV/FVC, and PEFR. The test was first explained and demonstrated to participants. Participants performed the test in a standing position with heads up. The measurement of the parameters was recorded as per cent of the predicted value.

The day before the test, the participants were edged not to consume alcohol, not eat large meals, and not to perform exercise in 4 hours, 2 hours, and 30 minutes, respectively. Just before the commencement of the lung volume measurements, the height and weight of each participant were measured with a stadiometer without their shoes using standard techniques. In performing the test, each participant was first asked to loosen any tight clothing, before instructed to connect to the vitalograph. This was done by putting the sterile disposable mouthpiece fixed to the vitalograph into their mouths on the tongue with the lips holding it air tight. A nose clip was used to hold the nose. The participant was allowed to familiarise themselves with the system before they been instructed to breath in to fill the lungs to the maximum. Each participant conducted two test manoeuvres: the static lung volume and dynamic lung volume.

In the static lung volume manoeuvre, the participants, after filling the lungs, pause and emptied the lungs into the vitalograph for the measurement of static lung volume. Again, the participant was asked to completely fill the lungs to the maximum pause and forcefully empty the lungs for the measurement of the dynamic lung volume. Each manoeuvre was repeated three times.

All measurements were taken between the hours of (9:00 am-12:00 noon) to minimize diurnal variation^16^. The precise technique of executing various lung function tests for the study was based on the operational manual of the instrument. The instrument was calibrated daily and operated within the ambient temperature range of 26-28°C.

### Data Analysis

The field data were entered directly into Microsoft Excel 2010 and analysed using GraphPad Prism 8.4.6. The level of statistical significance was set at a p-value <0.05 and statistical analysis was conducted using the student t-test to compare the mean lung function value between woodworkers and non-exposed workers (control) and between the woodworkers (carpenters and machine operators). The Fisher exact test was used to determine the frequency of respiratory symptoms at the workplace among wood workers and non-exposed workers. The ordinary one-way ANOVA was used to determine the frequency of respiratory symptoms at the workplace among woodworkers (carpenters and machine operators) and non-exposed workers. In addition, Pearson correlation was used to determine the relationship of the lung function values with years of work of the participants.

## Ethical approval

Approval for this research was granted by the Committee on Human Research, Publication and Ethics (CHRPE) of the School of Medical Sciences of Kwame Nkrumah University of Science and Technology (KNUST) and Komfo Anokye Teaching Hospital (KATH) Kumasi, Ghana (Ref: CHRPE/AP/304/15). In addition, permission was obtained from the Sokoban Wood Workers Association as well as the KNUST Primary and Junior High School and the KNUST Security Service for their staff participation. Informed written consent was also obtained from each participant before being enrolled in the study. The nature, purposes, and procedures of the study were explained to the association leaders and participants before seeking their approval.

## Results

### Demographic Indices of the Study Participants

The study selected 195 eligible participants comprising 96 woodworkers and 99 non-exposed workers (controls). A t-test comparison of the demographic data of the woodworkers and controls showed significant differences between the mean age and years of employment of the woodworkers and controls (p=0.0001). The woodworkers were much younger and had worked for a longer period than the controls. However, there was no significant difference in the mean height and weight of the two groups.

### Ambient air, wood dust

The particulate matter (PM) measured was greater than 2.5µm. Levels of inhalable wood dust particles measured at the four sites ranged between 0.005 and 0.102 mg/m^3^ for the machine operators and 0.003 – 0.882 mg/m^3^ for the carpenters. There was an average ambient temperature of 33°C and a mean relative humidity of 68%. Minimum exposure levels were recorded when the workers were not engaged on the machines, while the maximum exposure levels were recorded when the woodworkers were actively engaged on their machines.

### Respiratory Symptoms Reported by the Woodworkers and Controls at Their Workplace

The Prevalence of respiratory symptoms among woodworkers (carpenters and machine operators) compared with non-exposed workers (controls) at their various workplaces is shown in Table 2, The prevalence of all respiratory symptoms reported at the workplace was significantly higher in the woodworkers than the controls at (p<0.05). None of the controls studied reported of any of the listed respiratory symptoms at the workplace.

**Table 2:** Respiratory Symptoms among woodworkers and controls at their work place were compared

Symptoms	Controls % n=99	Carpenters % n=69	Adjusted p-value	Machine operators % n=27	Adjusted p-value
**Wheeze**	0	7	0.1313	37	0.0001
**Sneeze**	0	78	0.0001	96	0.0001
**Cough**	0	48	0.0001	52	0.0001
**Dry cough**	0	10	0.0258	30	0.0001
**Wet cough**	0	42	0.0001	44	0.0001
**Chest pain**	0	48	0.0001	37	0.0001
**Frequent fever**	0	47	0.0001	48	0.0001
**Breathlessness at rest**	0	14	0.0003	7	0.3097
**Breathlessness at work**	0	30	0.0001	7	0.4754
**Loss of voice**	0	32	0.0001	37	0.0001
**Rhinitis**	0	71	0.0001	70	0.0001

P-values; p<0.05

Rhinitis was prevalent among most of the woodworkers at the workplace. In addition, sneezing, wheezing, dry cough, wet cough, frequent fever, and loss of voice were comparatively more prevalent in machine operators than carpenters. While, chest pain, breathlessness at rest, and breathlessness at work were more prevalent among the carpenters.

### Respiratory symptoms in masked and unmasked woodworkers

A greater number of woodworkers (69) reported of not using a nose mask at the workplace. These were found to have a high prevalence (87%) of respiratory symptoms at the workplace. The woodworkers who reported of using a nose mask (27) were found to have a lower prevalence (74%) of respiratory symptoms at the workplace. However, it was only in wheezing (p=0.045), dry cough (p=0.007), and breathlessness (p=0.004) at work, that the prevalence were significantly higher in the unmasked woodworkers.

### Prevalence of Respiratory symptoms vacation

A small proportion of woodworkers 3% reported of persistent respiratory symptoms during vacation. While most of the woodworkers, 97% reported no respiratory symptoms on vacation. All controls reported no difference in their respiratory health during vacation.

### Comparison of Lung Function Indices between Woodworkers and Controls

The student t-test was used to compare the percentage predicted values of the lung function indices between the woodworkers and controls. The mean VC and FVC values were significantly reduced in the woodworkers compared with the controls. In addition, the mean FEV1 value was also reduced in the woodworkers compared with the controls, however, the difference was not significant. The mean FEV1/FVC and PEFR values were rather higher in the woodworkers than the non-exposed workers, although the differences were not significant (Table 4)

**Table 4:** Compared Lung Function Indices between Woodworkers and Non-Exposed Workers

Parameters	Woodworkers n=96 Mean (± SD)	Non-exposed workers n=99 Mean (± SD)	T	p-value
**VC**	88.3 ± 18.61	94.50 ± 11.62	2.94	0.0041
**FVC**	92.98±18.19	97.43±13.23	2.04	0.0438
**FEV1**	98.67±21.61	99.77±14.92	0.45	0.6553
**FEV1/FVC**	85.74±12.57	82.56±8.227	1.97	0.0516
**PEFR**	78.09±24.63	77.18±22.50	0.28	0.7763

P-values; p<0.05, SD: Standard deviation

### Correlation of lung function indices with years of work by woodworkers and controls

Table 5 presents the relationship between the lung function parameters with years of work by woodworkers and controls. The FEV1/FVC of the woodworkers showed a significant negative correlation with the years of work. Although the VC, FVC, FEV1, and PEFR of the woodworkers showed a positive non-significant correlation with their years of work. However, in the controls all lung function parameters showed a negative non-significant correlation with the years of work.

**Table 5:** Correlation of lung function indices with years of work by woodworkers and controls

Parameters	Woodworkers Years of work r (p-Value)	Control Years of work r (p-Value)
**VC**	0.1905 (0.0644)	-0.1082(0.2866)
**FVC**	0.0292 (0.7788)	-0.1709 (0.0907)
**FEV1**	0.0133 (0.8979)	-0.0950 (0.3495)
**FEV1/FVC**	-0.3699 (0.0002) ***	-0.0465 (0.6474)
**PEFR**	0.0205 (0.8436)	-0.1712 (0.0903)

P-values; p<0.05

### Proportions of woodworkers with lung function values below threshold

Greater proportions of the woodworkers had lung function values above the percentage predicted threshold values for all parameters (VC – 66%, FVC – 79%, FEV1 – 86%, FEV1/FVC – 94%) except PEFR (49%). However, the proportion of woodworkers with lung function values below the threshold for VC, FVC, and FEV1 were significantly lower (p<0.0001) compared with the controls. There was no significant difference between the proportions of woodworkers and controls for FEV1/FVC values below threshold.

## Discussion

### Level of Wood Dust Exposure

The present study sought to assess the pulmonary function of woodworkers exposed to mixed tropical hardwood dust in Kumasi, Ghana, where most of the woodworkers work without nose masks. The level of inhalable wood dust at the workplace measured in this study was in the range of 0.003 – 1.02 mg/m^3^. This was found to be lower compared to the documented permissible exposure level of countries such as the USA, European Union, Australia, and New Zealand (0.5 mg/m^3^ - 5.0 mg/m^3^). It was also found to be within the lower limits or below some documented exposure levels at which respiratory symptoms have been recorded in other studies. Gripenback et al. 2005^2^ reported various respiratory symptoms in woodworkers exposed to wood dust levels ranging from 0.2 – 7.4 mg/m^3^. Bohadana et al.^3^ observed bronchial hyper-reactivity in woodworkers exposed to dust levels between 2.96–12.74 mg/m^3^. In addition, Ennin^4^ reported the high frequency of respiratory symptoms in woodworkers exposed to dust levels ranging between 0.19 - 0.71 mg/m^3^. Our study was carried out in a region with a high annual relative humidity of 75%, In addition, all shops had high-rising concrete pillars supporting the roofing to improve ventilation. These may have contributed to the reduced atmospheric and ambient air wood dust concentration and therefore, reduced inhalable dust and exposure level. High humidity damps the inhalable wood dust and thus increases its weight causing it to fall under gravitational force. The present study suggests that high relative humidity may contribute to a low level of wood dust exposure.

### Prevalence of Respiratory Symptoms in Woodworkers

Respiratory symptoms in woodworkers are often initiated by irritant stimulation and an inflammatory reaction of the airways, which may result from an allergic response. In the present study, a high prevalence of respiratory symptoms at the workplace was observed in the woodworkers. The prevalence of one or more respiratory symptoms at the workplace was between 7% - 96%. This result is consistent with other studies that have reported a high frequency of respiratory symptoms in woodworkers^5^,^10^ - ^12^,^14^,^17^

In our study, the majority of the woodworkers did not use nose masks while at work, which exposed them to much inhalable dust from the ambient air. They reported never using a nose mask, nor did they use any form of protective gear. The few who reported using a nose mask only used it to work and removed it while waiting for the next task. The reason was that the available nose masks were not appropriate for woodworkers. They, therefore, find it very uncomfortable for prolonged use and thus prefer wearing it only when actively working. Comparing the prevalence of respiratory symptoms among woodworkers using the nose mask and those not using it at all, the study observed that those woodworkers who did not use the nose mask at all had a higher prevalence (87%) of respiratory symptoms. The low prevalence among those who wore the nose mask can be attributed to the fact that they removed it soon after the task was completed and thus exposed themselves to ambient air and wood dust. This suggests that woodworkers who do not wear nose masks at all are more exposed to inhalable wood dust and may be more vulnerable to respiratory disease. This is consistent with the suggestion of Liou et al.^1^ that a high level of wood dust exposure by woodworkers leads to occupational respiratory hazards.

In addition, the study observed a high prevalence of rhinitis among woodworkers (71%). This is consistent with the findings of Jacobsen et al.^18^ and Soongkhang^10^. This finding can be attributed to chemical or mechanical irritation from the wood dust in the ambient air, which may have stimulated the respiratory symptoms in the woodworkers. However, some of the respiratory symptoms had a low prevalence in woodworkers (7%). This is also consistent with the findings of Bohadana et al.^3^, Borm et al.^19^, and Fransman et al.^13^, who found few or none of the woodworkers in their studies with respiratory symptoms. This study also observed that most of the woodworkers reported discontinuous respiratory symptoms during vacation. This is consistent with the finding of Boskabady et al.^20^, who observed that most respiratory and allergic symptoms in carpenters significantly increased during work compared to their rest period.

### Lung Function Indices Compared Between Wood Workers and Non-Exposed Workers

Reduction in lung function index is a characteristic of respiratory defects, including restrictive, obstructive, and combined defects. The restrictive defect, such as in pulmonary fibrosis or oedema, is characterised by a reduction in VC, FVC, and FEV1 below the threshold values. An obstructive defect such as asthma is characterised by a reduction in FEV1 and FEV1/FVC below the threshold value. Reduced PEFR is a characteristic of narrowing of the airways and weakness in the expiratory muscle^21^,^22^.

In the present study, the majority (>50%) of the woodworkers and controls had their lung function values above the reference threshold values, except for PEFR. This is consistent with previous studies by Borm et al.^19^ and Baran et al.^23^, who found no negative effects of wood dust on the lungs of the woodworkers they studied. This finding suggests that most of the woodworkers may not have respiratory defects, due to the low ambient air wood dust concentration and the few who use nose masks. The study also observed that the Sokoban Wood Village had a young workforce that had not been on the job long enough to manifest a respiratory defect due to prolonged exposure to wood dust. In addition, older woodworkers may have stopped working as woodworkers and left the industry.

This study also observed a significant reduction in the lung function of the woodworkers, particularly in the VC and FVC, compared with the controls. Other studies have reported significant reductions in lung function indices in woodworkers compared to controls^10^-^12^. Ennin^4^, in a study at the Accra Timber Market, reported a significant reduction in the lung function indices of woodworkers.

However, there was a significantly high proportion of woodworkers with lung function values above the threshold (although less than the controls). This implies that a smaller number of the woodworkers were below the threshold values. When classifying those with reduced lung function values below the threshold, some of the woodworkers may be classified as having restrictive and obstructive lung defects. This finding suggests that not all woodworkers may be vulnerable to having a respiratory defect. In another study, the majority of the woodworkers were found to have lung function values above the threshold^4^.

The study also observed a significant negative correlation of FEV1/FVC with years of work with the woodworkers. This finding suggests that the longer one stays in the woodworking business, the lower the FEV1/FVC becomes. Reduction in FVE1/FVC below the threshold is a characteristic of an obstructive defect. This suggests that the longer the years of work as a woodworker, the higher the tendency of getting an obstructive defect.

The variation in the results of different studies may be attributed to either the different wood species and/or the various levels of wood dust exposure by woodworkers. The present study, therefore, suggests that a low level of wood dust exposure and the use of a nose mask may contribute to a low prevalence of respiratory symptoms and a smaller number of woodworkers with reduced lung function status.

## Conclusion

This study revealed that woodworkers exposed to mixed tropical hardwood dust, mostly without personal protective equipment, may have a high prevalence of rhinitis but discontinuous respiratory symptoms when away from the workplace. The study further revealed that woodworkers with lung function values below the reference threshold may have restrictive or obstructive respiratory defects, while those who spent longer years in business may end up with an obstructive defect.

## Study Limitation

The study was faced with a number of shortcomings which should also be considered in further studies. Some of these shortcomings were as follows:

Small sample size: this might have accounted for the large number of wood workers having most lung function parameters above the threshold values. This might have influenced the weak correlation between lung function and other variables such as duration of exposure.
The participants in this study, as in most cross-sectional studies, may represent a survival population, so workers with more disabling symptoms might have changed jobs. This could have resulted in the inconsistency observed in respiratory symptoms, association with lung function defects and duration of exposure.

## Figures and Tables

**Table 1:** Demographic Indices of the Wood Workers and Non-Exposed Workers

Variables	Non-exposed workers	Woodworkers		
	n =99 (mean ± SD)	n = 96 (mean ± SD)	T	p-value
Age	42.24 ± 9.06	36.28 ± 12.23	3.88	0.0001 *
Height	169.0 ± 5.85	170.3 ± 7.34	1.35	0.178
Weight	70.15 ± 11.27	69.01 ± 9.4	0.76	0.446
Years of Employment	11.96± 7.63	17.61±11.80	3.98	0.0001*

P-values in parentheses

* p<0.05 SD: Standard deviation

**Table 3:** Prevalence of respiratory symptoms in masked and unmasked woodworkers

Symptoms	masked n=27	unmasked n=69	% of ww masked	% of ww unmasked	T	p-value
**Wheeze**	1	14	4	20	2.035	0.045*
**Sneeze**	20	60	74	87	1.525	0.131
**Dry cough**	0	16	0	23	2.735	0.007*
**Wet cough**	11	30	41	43	0.241	0.809
**Frequent fever**	11	34	41	49	0.747	0.457
**Breathlessness at rest**	1	11	4	16	1.636	0.105
**Breathlessness at work**	3	29	11	42	2.992	0.004*
**Lose of voice**	9	23	33	33	0	>0.999
**Rhinitis**	16	52	59	75	1.564	0.121
**Chest pain**	9	34	33	49	1.412	0.161
